# Elimination of ASFV via Precise Culling in a Large-Scale Breeding Herd in China: A Field Experience

**DOI:** 10.3390/ani15172521

**Published:** 2025-08-27

**Authors:** Xingqian Du, Yuan Liu, Lianmao Duan, Shih-Yi Tsai, Joseph P. Yaros, Fangzhou Wu

**Affiliations:** 1 Pipestone International Management, Pipestone, MN 56164, USA; xingqiandu43@gmail.com (X.D.); hunter142234@163.com (Y.L.); duanlianmao@163.com (L.D.); franz306@hotmail.com (S.-Y.T.); 2 Pipestone Veterinarian Service, Pipestone, MN 56164, USA; joseph.yaros@pipestone.com

**Keywords:** ASFV, elimination, low-virulence strains

## Abstract

This field study presents an elimination strategy for African Swine Fever Virus (ASFV), particularly low-virulence genotype II strains, in a large-scale breeding herd in China. Since its introduction to China in 2018, ASFV has spread rapidly, causing significant economic losses. The emergence of low-virulence strains in 2020 further complicated the efforts against this virus. We describe a case study on a farm with 6000 sows in eastern China. Upon ASFV detection, pig movements were ceased immediately, strict biosecurity protocols were implemented, and an epidemiological investigation was conducted to identify high-risk zones. Pigs that tested positive or within high-risk zones were culled, followed by extensive disinfection of exposed areas and whole-herd PCR screening. Injection of a foot and mouth disease vaccine was utilized to stimulate virus excretion of latent infections for better detection. Four rounds of whole-herd tests were conducted to confirm the elimination of ASFV. This case study highlights the importance of timely monitoring, rapid culling, thorough disinfection, and enhanced biosecurity as an effort to control ASF outbreaks in a low-impact and more cost-efficient manner, so called “tooth extraction.”

## Simple Summary

African Swine Fever (ASF) is a severe disease that threatens the pig farming industry, causing severe economic losses. This paper shares experiences in eliminating the ASF virus, specifically a low-virulence genotype II strain, from a large-scale breeding herd in China by employing strict biosecurity measures, extensive sampling and testing, and precise culling of infected and high-risk pigs. In contrast to traditional depopulation, precise culling provides a low-impact and cost-efficient strategy to control and eliminate the ASF virus in commercial sow farms.

## 1. Introduction

African swine fever (ASF) is an acute, febrile, and infectious disease caused by the African swine fever virus (ASFV). This disease affects both domestic pigs and wild boars, with soft ticks serving as the primary transmission vector and reservoir host [1]. The clinical symptoms of ASF range from acute to subacute and chronic forms, with major manifestations including high fever, cyanosis of the skin, extensive hemorrhage in internal organs, respiratory distress, and neurological symptoms, and the mortality rate can reach as high as 100% [2]. ASF has inflicted tremendous losses on the worldwide pig farming industry. Currently, there are no commercially available vaccines against ASF, so spread of the disease can only be controlled by locking down epidemic zones and depopulating the site [3].

African swine fever was first identified in Kenya in 1921, initially occurring in countries south of the Sahara in Africa [4]. The ASFV genotype I was first introduced from West Africa to Europe in 1960, marking the beginning of its spread beyond the African continent [5]. In 2007, ASFV genotype II emerged from East Africa and rapidly spread across Europe, causing widespread outbreaks [6]. In 2018, this virus strain was introduced into China and 10 other Asian countries [7]. The consequences of an ASF outbreak in China have been catastrophic, both for the domestic economy and for international trade [8]. On 3 August 2018, the World Organization for Animal Health (WOAH) announced the initial detection of ASFV in Shenyang City, located in China’s Liaoning Province [9]. The strain responsible for this outbreak belonged to genotype II [10]. Despite the emergency measures implemented by the Chinese government, including strict quarantine and culling protocols, the ASFV outbreak subsequently spread rapidly to all 31 provincial-level administrative regions across the country [11]. The virus strain was isolated and identified as genotype II, sharing high homology with the Georgia-07 strain, which is known to cause acute infections and is associated with 100% mortality in field pigs [10]. With the prevalence of ASF in China, epidemiological surveillance of circulating strains in 2020 revealed the emergence of not only highly virulent genotype II strains but also low-virulence genotype II strains [12]. Whole-genome sequencing indicated a deletion in the EP402R gene, which encodes the CD2v protein, resulting in the loss of hematopoietic adhesion capability in these low-virulence strains [13]. These low-virulence strains can cause mild mortality in pigs at high infectious doses, while at low doses they lead to persistent, non-fatal, subacute or chronic diseases. The long incubation period and high transmissibility of these low-virulence strains make early diagnosis more challenging [12].

A commercial vaccine against ASF (ASFV-G-∆I177L) has recently became available, but it has not been approved in China [14,15]. Consequently, culling of the infected animals along with the implementation of strict sanitary measures remains the only options to control this devastating disease in China. With the persistence of ASF outbreaks, a precise culling approach, also known as “tooth extraction,” has been widely implemented [16,17]. Compared to diseases like Porcine Reproductive and Respiratory Syndrome (PRRS) and influenza, the spread of ASFV within pig populations is relatively slow, primarily occurring through direct contact [3]. Therefore, if infected animals can be promptly identified and properly disposed of along with rigorous disinfection of the environment during the early stages of virus transmission, effective suppression of the virus’s proliferation throughout the entire herd may be achieved. In current Chinese sow farms, pigs are predominantly housed in individual stalls with limited physical contact among them. Strict biosecurity measures implemented at the initial stage of disease occurrence can, to a certain extent, slow down or even block the virus’s transmission. These factors serve as the basis for potential success in eradicating ASFV from farms.

Although the “tooth extraction” strategy has proven to be effective in eliminating high-virulence ASFV infections in sow farms, its application becomes more challenging when dealing with low-virulence strains due to their longer incubation periods and more concealed transmission characteristics [18,19]. In this case study, we present a successful “tooth extraction” operation against a low-virulence genotype II strain of ASFV in a large-scale breeding herd in China.

## 2. Materials and Methods

### 2.1. Ethical Review

Following the Pipestone Research IACUC review, it was determined that an ethical review of the farms reported in the field case study was not needed, as no special treatment or procedure beyond established production protocol was involved. In addition, the farm owner granted Pipestone International Management permission to use site data for research and publication.

### 2.2. Farm Description

**Figure 1:**
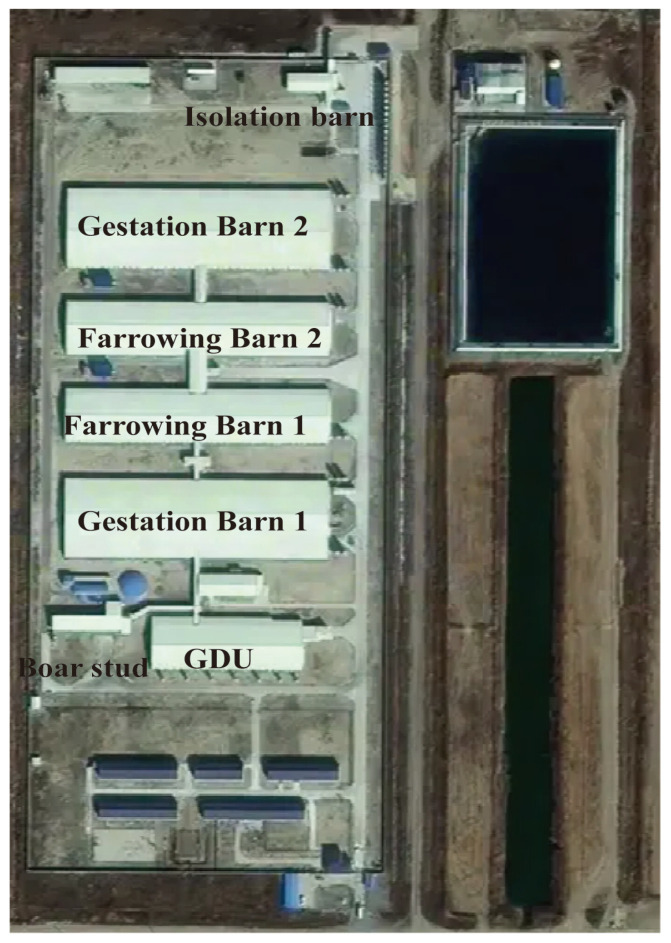
Layout of the farm.

The production site described in this case study is located in a pig-dense zone in eastern China (Shandong Province) with a stocking capacity of 6000 sows. It is equipped with automatic feeding and drinking systems along with comprehensive biosecurity designs. The farm consists of two gestation barns, two farrowing houses, one gilt development unit (GDU), one boar stud, and one isolation barn, as shown in Figure 1.

### 2.3. Biosecurity

Given the high pig density in the region, the sow farm has implemented strict biosecurity protocols since the start of its operations, as previously described [20]. Frequent entries by employees or non-essential visitors are minimized. Personnel entering the farm must quarantine in an off-site dormitory for two days before being allowed into the production zone. Employees and visitors entering the farm (whether to the production zone or the dormitory) are required to shower at the farm entrance and have samples collected from their hands, coveralls, and boots for biosecurity records. Only essential personal belongings are permitted to be brought into the dormitory after disinfection, but they are not allowed into the production zones. If entry into the production zones is necessary, showering is required upon both entering and exiting at the pig shed entrance. Supplies, including food ingredients for the cafeteria, must be cleaned and disinfected before entering the farm. The farm internally produces replacement gilts and has an on-site boar station to avoid the introduction of external semen.

### 2.4. Disease Monitoring

**Figure 2:**
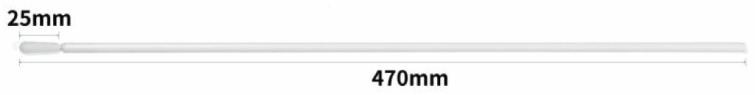
Disposable throat swab used for sampling.

Early detection of ASFV in an outbreak is the key to successful “tooth extraction.” A routine monitoring protocol is implemented as follows:

- Boars for semen collection: Sample boars one day before semen collection; all boars should be sampled at least once a week. If any boar shows abnormal behavior, it should not be utilized for semen collection and it needs be sampled and tested immediately.
- Pig with abnormal behavior: Pigs with signs of fever, anorexia, abortion, vomiting, lameness, or any abnormal appearance should be identified and sampled for an ASFV test. A throat swab sample, as a preferred method of sampling for ASFV [21], is collected using a long swab, as shown in Figure 2. Technicians should wear disposable long-arm gloves when sampling. Additional environmental samples are collected around the abnormal pig using cotton swabs and pooled in the same sampling tube with the throat swab. Abnormal pigs sampled per day typically account for approximately 1.5–2% of the herd as routine monitoring. Aborted sows should be sampled for three consecutive days and then tested again a week after to confirm a negative test result. Treatments for abnormal pigs are held until the day after the PCR results are available to avoid potential spread of disease during the treatment procedure.
- Gilt entry: Before integrating gilts from GDU into the herd, cotton rope samples from each gilt pen are required. Only transfer gilts after negative results are confirmed.
- Nurse-off sows: Sows that stopped nursing with feed refusal must have two negative tests within five days before they can be removed from farrow crates. Sows with insufficient milk production but with normal feed intake can be transferred after one negative test result.
- Environmental sampling: The non-production area should be sampled at least once a week, including the cafeteria, office, showers, dormitory rooms, etc. Environmental samples from inside each barn should be collected twice a week, including the euthanasia device, electronic carcass transporter, and boar carts for heat checking, etc. Dust samples from inside feed bins are collected to monitor the risk of virus transmission via feed. Cotton gauze is hung next to the barn air intake for environmental air monitoring.
- Mortality disposal: Any dead pig is assumed to be ASF-positive and should not be moved or disposed until the test results are available. This is to avoid potential transmission through contact.

During the ASF low-risk season, samples are pooled in 10:1 ratio for PCR testing. However, when the disease risk is elevated (e.g., winter, rainy season, regional outbreak, etc.), the frequency of sampling should be increased accordingly and the pooling size is decreased to 5-in-1 for testing unless in special cases where single testing is required.

### 2.5. Diagnosis

A positive case of ASF is typically confirmed by detection of antigens or antibodies. As previously described, antigens are detected using a quantitative polymerase chain reaction (qPCR) test [22]. Samples for detection include serum and oral fluid. In brief, 200 µL of serum or oral fluid are subjected to automated DNA extraction (MultiEX032, Shengce Biotechnology Co., Ltd., Changsha, Hunan, China). Subsequently, 5 µL of the extracted nucleic acid is used for qPCR testing. The reaction is performed using a Step One Plus instrument (ABI, Applied Biosystems, Waltham, MA, USA) using the RealPCR* ASFV DNA Test kit (99-56020, IDEXX Laboratories, Inc., Westbrook, ME, USA), according to the manufacturer’s protocol. The test is carried out under the following conditions: 1 cycle at 95 °C for 1 min, and 45 cycles at 95 °C for 15 s and 60 °C for 30 s; a sample with cycle time (Ct) value less than 40 is considered ASFV-positive. A low-virulence genotype II strain is confirmed by using an ASFV (p72/CD2v gene) Dual Real-Time PCR Test Kit (Lijian Bio-Tech Co., Ltd., Qingdao, China), according to the manufacturer’s protocol: 1 cycle at 95 °C for 20 s, and 40 cycles at 95 °C for 10 s and 58 °C for 20 s; a sample with a Ct value less than 35 is considered positive for the p72/CD2v gene. When a positive sample is determined, the original sample is retested, as well as re-sampling of the suspect pig, to rule out false positivity.

## 3. Results

### 3.1. ASF Outbreak

**Figure 3:**
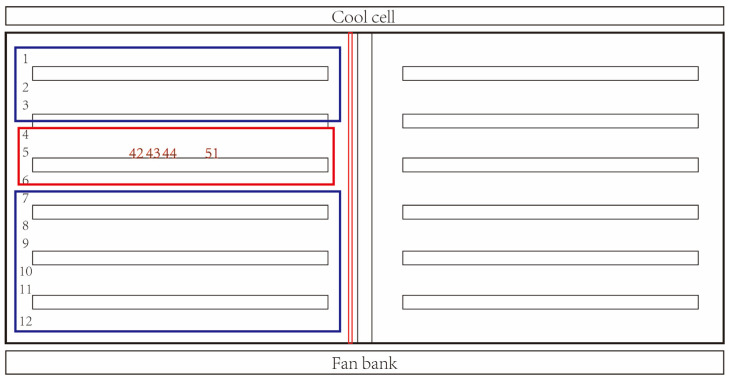
Layout of the gestation barn 1. The red numbers represent the crates where positive pigs are located. The blue frames represent the medium-risk zone. The red frame represents the high-risk zone.

Infection with ASFV was first detected on 2 February 2023. A gestating sow exhibited reduced feed intake and tested positive for ASFV. The sow was located at row 5, crate 43, in gestation barn 1 (G1-5-43) and was in the mid-stage of gestation (Figure 3). No other abnormality was recorded for this sow in the recent period. The Ct value of the qPCR test was 29.97. Identification of strain type was also performed and confirmed the infection virus as a low-virulence strain with deletion of the CD2v gene (Table 1).

### 3.2. Epidemiological Investigation

Following the confirmation of infection, all pig movements within the farm were immediately halted, and an on-site epidemiological investigation was initiated to identify high-risk zones. The considerations for risk zoning included the following:

- Was the infected pig recently moved within the barn? What was the route of movement?
- Were there personnel activities (e.g., breeding, health checking, treatment, body condition checking, etc.) or any equipment that had been in close contact with the index pigs?
- Location of the index pig within the barn (a map of crate layout is needed). How many pigs were housed in proximity, especially with a shared feed trough?
- Identify alleys in the neighborhood and public zones with frequent people and pig traffic.

Based on the epidemiological investigation, zones of high risk where direct or indirect contact with the infected sow was possible was determined, including the row where the infected sow was located and the two rows adjacent (rows 4, 5, and 6; Figure 3). The high-risk zone was considered rather limited because the index sow was in the mid-stage of gestation with no other abnormal records and had not been recently moved nor subjected to boar contact. However, given that the virus of infection was a low-virulence virus strain with CD2v deletion, known for a long incubation period and higher transmissibility than wild-type ASFV, a relatively large zone of medium-risk was considered, including the entire east half of gestation barn 1.

On the same day of ASF detection, throat swab samples from a total of 312 pigs from the high-risk zone were collected. Samples were pooled in a 3:1 ratio for ASFV testing. Meanwhile, environmental samples were taken from high-traffic work zones, such as the office entrance and gestation barn 1 entrance, for ASFV detection. Three pigs were confirmed positive, all located within the same row as the first positive sow (row 5, crates 42, 44, and 51; Table 2). All environmental samples tested negative.

### 3.3. Precise Culling

The culling process started immediately after the scope of infection and risk zones were determined. Again, due to the fact the infection was caused by a low-virulence ASFV strain, a relatively stringent culling approach was implemented. This decision was also made with consideration of the layout of the farm, where gestation barn 1 is located between the barn office (main entrance) and other gestation and farrowing barns (Figure 1), so the risk of spreading virus is high due to ineluctable personnel traffic.

The four positive sows were euthanized (electrocution), placed in impermeable plastic bags, and moved out of the farm for incineration. A total of 1097 pigs from the whole high- and medium-risk zones (half of gestation barn 1) were culled within 24 h, which accounted for about 18% of the breeding inventory.

During the culling process, a strict biosecurity protocol was implemented to minimize potential transmission of the pathogen. Plastic sheets were applied to physically isolate the west and east sides of gestation barn 1. Alleys were covered by plastic sheets during pig movements to avoid cross contamination and for easier disinfection afterward. Additional sets of exhaust fans on the east side of gestation barn 1 were turned on. This was an effort to maintain higher negative static pressure in the culling zones, minimizing movement of potentially contaminated dust or aerosols from east to west side of the barn.

After culling the positive and high-risk pigs, three rounds of thorough cleanings and disinfections of the east section of the gestation barn 1 were conducted using a 3% sodium hydroxide solution. Feed lines, feed boxes, roof, and fan louvers were disinfected with Virkon (potassium peroxymonosulfate). Additional disinfection with hypochlorous acid was applied to the crates where the positive pigs had been housed to eliminate any remaining ASFV nucleic acid material. After cleaning and disinfection, the east section of gestation barn 1 was thoroughly dried and left vacant for three months.

If passing through gestation barn 1 was necessary to reach farrowing barns and gestation barn 2, staff were required to don an extra layer of plastic coverall, shoe covers, and gloves, and shed them after passing through. The same precautions were taken on the way back.

### 3.4. Whole-Herd Screening

**Figure 4:**
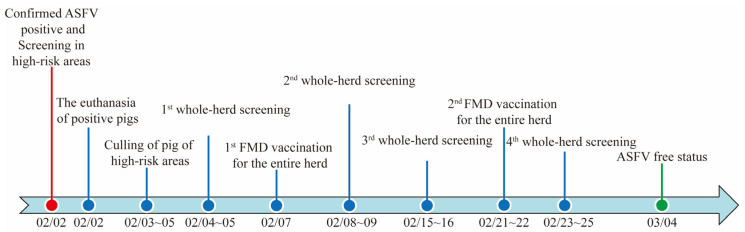
Timeline of African Swine Fever outbreak, precise culling, whole-herd screening, and elimination.

Following the culling of infected and at-risk pigs, whole-herd sampling and testing were carried out repeatedly to screen for additional infected pigs. Throat swab samples were collected from each pig following the order of the gestation crates and were pooled in a 3:1 ratio for testing. To prevent cross-contamination during sampling, wearing sterile disposable gloves was required, which were replaced every 3 samples. Prior to the second and fourth rounds of screening, a foot and mouth disease (FMD) vaccination was given to each pig. The adjuvant present in this vaccine creates a stress response in the recipient population, completed with the aim of stimulating ASFV shedding by latent pigs for better detection. With all pigs testing negative for four rounds, successful elimination of ASFV from the farm was declared. The timeline of the major action events during this ASF case is provided in Figure 4.

### 3.5. Downstream Farms

In the winter (November–February), when the risk of ASF is high in this region, an empty nursery site is always prepared as a dedicated reserve facility. Should an outbreak occur at the sow farm, weaned pigs would be immediately diverted to this alternative site to avoid transmission to downstream farms. Concurrently, rigorous surveillance protocol would have been activated across all downstream operations. In this case, during 21 days of stringent surveillance, no positive sample was found from the original nursery receiving piglets from the sow farm. Similarly, no weaned pig at the reserve site tested positive during 28 days of intensive monitoring. These results verified that the farrowing house at the sow farm was intact during the outbreak.

## 4. Discussion

Due to high pig density, the prevalence of ASF outbreaks has been historically high in the region. In this case, a farm located 2.6 km west of the present farm reported an outbreak one week prior to our first detected infection with the same ASFV strain, which could have been the index case of this regional outbreak. Seven days later, a farm located 3 km southeast of the present farm also reported an infection with the same virus. It was suspected that ASFV might have been transmitted via dust aerosols carried by strong winds in February. A biosecurity breach was also identified on the farm, as a bird’s nest was found inside the feed bin cap, which could have been a vector of virus transmission.

Once ASFV is detected, a prompt investigation into the infected individual is critical for understanding the scope of infection. Based on the timing of detection, pen or crate location, recent pig movement history, and the Ct value of the first positive sample, zones of different risk levels can be determined. Pigs with direct contact and areas with any movement history of the infected individual should be designated as high-risk zones. High-risk zones should be physically cordoned off, and under no circumstances should anyone be allowed to enter without the presence of trained personnel. Other zones outside the high-risk zone should be categorized according to their biosecurity levels. Under normal circumstances, in the biosecurity hierarchy of a sow farm, the biosecurity level decreases in the order of boar stud, nursery, GDU, farrowing house, gestation barn, isolation barn, and loadout chute. In the event of an ASF outbreak, protection of the boar stud is prioritized due to the high economic loss of culling. The boar stud often has a relatively low risk of infection given the limited personnel and pig movement. The GDU comes next, as developing gilts are housed in group pens, so the virus will spread fast if an outbreak occurs. In the present case, the first infected pig was in the mid-stage of gestation with minimal pig and personnel contact and infection was detected rather quickly after its decrease in feed intake, so the scope of infection and contamination was relatively small.

Extensive sampling is needed to understand the status of virus transmission. Areas where personnel have frequent contact are focused, such as barn door handles, office, supply storage, mortality disposal, exhaust fan outlets, etc. Before the infection scope is determined, all production activities are halted that involve direct contact with pigs, except for water and feed provision, health chore, mortality disposal, and disinfection. This period is referred as the “silent period.” The duration of the silent period depends on the size of the farm and operation model (e.g., continuous vs. batch farrowing), and it should be adjusted dynamically as the scope and status of infection become clearer. Generally, the longer the silent period can be maintained, the less spread of the epidemic due to pig movement, but the bigger the economic loss due to suspended production. Maintaining a long silent period can be challenging due to tight production schedules, especially for farms with continuous farrowing operations. If an extended silent period is needed, the following measures can be considered: (a) prioritize the sampling of animals where movement is urgent (e.g., due to farrowing, need for treatment, etc.) and multiple tests are required; (b) induced abortion may be necessary for sows due to farrowing to avoid movement, and aborted piglets and afterbirth should be promptly collected by designated personnel; or (c) directly cull a batch of due-to-farrow sows.

Determining whether the infection was caused by a wild-type ASFV strain or a gene-deleted low-virulence strain is another factor that will dictate the subsequent handling plans. For farms infected with wild-type ASFV, a smaller culling size can be implemented because wild-type strains can be more quickly and precisely detected by throat swab samples and PCR tests. However, infection with a gene-deleted strain usually has a long incubation period with reduced pathogenicity, so it takes an extended period of time before symptoms are detected, resulting in a greater scope of infection. In addition, gene-deleted strain-infected pigs may shed virus intermittently [23,24], making it even harder to be detected during whole-herd screening.

Once a thorough epidemiological investigation is completed, precise culling of infected and at-risk pigs can be carried out. If the culling size is small, infected pigs should be euthanized and wrapped in a plastic sheet before being transferred to minimize cross-contamination. At the same time, pigs in the adjacent crates to the positive pig should be euthanized. For example, if the infected pig is in a gestation crate, five sows each to the left and right side of the same row should be euthanized, along with five sows each from the front and back rows. Preferred methods of euthanasia are the ones with minimal exposure of blood and oral fluids, which are highly infectious. If the number of pigs to be culled exceeds the capacity of being handled inside the barn, they need to be transferred off site for culling. Extra caution is needed when transferring high-risk sows. The alley behind the crates should be used to minimize mouth-to-mouth contact with other pigs. Prior to transfer, alleys and corridors should be disinfected in advance and covered with layers of plastic sheets to minimize contamination of the environment. After pigs have moved through, immediate and thorough disinfection of the entire traffic zones is required. We disinfected the back alley with a 3% sodium hydroxide solution and the front alley with a 1:100 Virkon solution (Potassium peroxymonosulfate). Disinfection should be performed for five consecutive days.

According to the World Organization for Animal Health (WOAH), the standard incubation period of ASF is generally 15 days. Le et al. (2023) indicated that, in small-scale groups, the longest incubation period for ASF can reach 19 days [25]. However, the actual incubation period may vary depending on the virus strain, host, and route of infection, typically ranging from 5 to 19 days [26], and can extend up to 21 days in extreme cases [27]. Since the virus strain involved in this outbreak was a CD2v gene-deleted strain, its virulence was lower than wild-type strains. Clinical symptoms were mild and less pronounced, and the shedding of virus by infected pigs occurred relatively late. Koltsov et al. (2023) found that deletion of the CD2v gene in the virulent ASFV Congo strain led to a delay in the onset of viremia in pigs; specifically, the virus titer in the blood of pigs infected with the ΔCongo-v_CD2v strain was significantly lower in the early stages of infection compared with those infected with the parental Congo-v strain [24]. This delay suggests that the CD2v gene plays a role in the early replication and spread of the virus within the host [24]. Sun et al. (2021) found that the incubation period of CD2v-deficient low-virulence ASFV strains isolated in 2020 ranged from 7 to 28 days, with a mean of 21 days [28]. In the present case, the last positive pig was detected on 2 February, and the final round of whole-herd screening was completed 21 days after detection of the last positive sample. Following the completion of the fourth whole-herd screening, enhanced surveillance reverted to routine protocols. ASF-free status was declared 32 days later, covering the maximum incubation period of ASF reported.

Use of the FMD vaccine before herd-wide screening has become a standard practice when handling low-virulence ASFV strain outbreaks, but, to our knowledge, has not been documented in previous literature. The mechanism of how the FMD vaccine facilitates viral shedding from sub-clinical infection remains unclear. The target cells of ASFV are mononuclear phagocytes, such as macrophages and dendritic cells [29]. The receptor for ASFV is currently unclear, but Sánchez-Torres et al. (2003) demonstrated that CD163 expression confers susceptibility to ASFV infection in porcine macrophages [30]. In inactivated FMD vaccine, traditional aluminum adjuvants promote M2 polarization of macrophages at the injection site by inducing high IL-4 expression [31]. The M2-polarized macrophages provide a more permissive intracellular niche for ASFV, as demonstrated by Sánchez-Cordón et al. [32]. The M2 macrophages, stimulated by anti-inflammatory cytokines, highly express the CD163 receptor, which in turn may facilitate infection with ASFV [32]. Meanwhile, it is also possible that non-specific immune functions of pigs, including the integrity of mucosal barriers, may be impaired in response to the FMD vaccine and, therefore, lead to ASFV shedding.

The attempt to use a precision culling strategy can be traced back to 2019 in China’s pig industry, and it has “matured” over the past years. Among the farms we operate, our first experience with low-virulence ASFV infection was in December 2020 with a herd of 6000 sows, where the whole herd was depopulated. In another attempt in December 2021, precision culling was first practiced in a 5600-sow farm. However, with limited knowledge about low-virulence ASFV strains, the detection of subacute infection was untimely and the precision of whole-herd screening was impaired by over-pooling samples for PCR tests, ultimately resulting in failure. With more developed action protocols and better biosecurity execution, our first successful “tooth extraction” with low-virulence ASFV was achieved in February 2022 in a 6000-sow farm, where 46.55% of the herd was culled. During the 2023–2024 disease season, we encountered a total of 3 outbreaks with low-virulence ASFV and all cases were successfully controlled by precise culling with a culling size less than 16% of the whole herd.

In general, precise culling offers significant cost advantages over whole-herd depopulation. A crude economic analysis of the present case was conducted in Table 3. Precise culling led to only 18.2% of inventory loss, so it decreased direct bio-asset loss by 87% and restocking costs by 18%. In addition, production was only seized for 32 days versus a minimum of 6 months for depopulation; therefore, the opportunity loss due to production interruption was drastically decreased. Together, under the assumed market scenario, precise culling only resulted in about 15% of the total economic loss compared with depopulation. However, it should be noted that a failed precise culling practice can be more costly than a timely depopulation due to its increased operation costs and prolonged production interruption.

## 5. Conclusions

Precise culling, or “tooth extraction,” provides an effective approach to contain the spread of ASFV within a farm, with reduced economic losses compared to depopulation. Our case of an ASF outbreak demonstrated the importance of robust biosecurity protocols in preventing disease introduction to sow farms, especially a meticulous routine monitoring program for early detection of infection. When a “tooth extraction” strategy is employed, a quick and detailed epidemiological investigation of the scope and status of infection is key to success, followed by prompt culling of high-risk pigs and thorough disinfection of affected zones.

## Figures and Tables

**Table 1:** Test results of the first positive sample and confirmation of low-virulence strain.

First Positive Sow	IDEXX ^1^	Lijian (p72/CD2v Gene) ^2^	
	**P72**	**P72**	**CD2v**
The 1st test of the original sample	28.27	33.47	Negative
The 2nd test of the original sample	29.97	32.38	Negative
The 1st test of re-sampling sample	27.36	30.89	Negative
The 2nd test of re-sampling sample	27.99	31.63	Negative

^1^ Polymerase chain reaction (PCR) test kit manufactured by IDEXX (USA). ^2^ Polymerase chain reaction (PCR) test kit manufactured by Lijian (Qingdao, China).

**Table 2:** The locations and Ct values of pigs testing ASFV-positive.

Location	CT Value
G1-5-42	36.08
G1-5-44	37.12
G1-5-51	40.99

**Table 3:** Cost comparison: “tooth extraction” vs. total depopulation for ASF control.

Category	Depopulation	Precise Culling
Animal loss ^1^		
Sow, n	6000	1097
Replacement gilt, n	2011	0
Piglet, n	8958	0
Boar, n	136	0
Total animal value, RMB	¥25,264,756	¥3,291,000
Mortality handling cost, RMB	¥81,470	¥10,970
Testing costs, RMB		
FMD vaccination (2 times)	-	¥28,200
Whole-herd PCR tests (4 times)	-	¥142,417
Cleaning & disinfection costs, RMB	¥85,000	¥17,000
Restocking costs, RMB ^2^		
Replacement gilt	¥15,000,000	¥2,742,500
Boar	¥408,000	-
Production interruption (opportunity loss)		
Wean pig (6 months throughput), n	74,520	13,625
Piglet profit loss, RMB	¥11,178,000	¥2,043,711
Total economic loss, RMB	¥40,839,226	¥6,232,087

^1^ Market values used in calculation: live hog = 14.2 RMB/kg, wean pig = 250 RMB/head, replacement gilt = 2300 RMB/head, boar = 3000 RMB/head. ^2^ Assumed restocking cost: gilt = 2500 RMB/head, boar = 3000 RMB/head.

## Data Availability

The original contributions presented in this study are included in the article. Further inquiries can be directed to the corresponding author.

## Abbreviations

The following abbreviations are used in this manuscript:

ASF	African swine fever
ASFV	African swine fever virus
WOAH	World Organization for Animal Health
HAD	Hematopoietic adhesion
PRRS	Porcine Reproductive and Respiratory Syndrome
PCR	Polymerase chain reaction
GDU	Gilt development unit
